# Nanobubbles and Fibroblast Growth: An In Vitro Study on Cell Migration and Proliferation

**DOI:** 10.7759/cureus.74775

**Published:** 2024-11-29

**Authors:** Mei Sanno, Junya Kusumoto, Hiroto Terashi, Shunsuke Sakakibara

**Affiliations:** 1 Department of Plastic Surgery, Kobe University Graduate School of Medicine, Kobe, JPN; 2 Department of Oral and Maxillofacial Surgery, Kobe University Graduate School of Medicine, Kobe, JPN

**Keywords:** microbubble, nanobubble, nano particles, ozone therapy, ­wound healing, wound infection

## Abstract

Nanobubbles are studied for their unique properties and possible applications in wound healing processes. This study investigates the effects of hydrogen (H₂), oxygen (O₂), and ozone (O₃) nanobubbles on fibroblast migration and proliferation using *in vitro* scratch wound healing assays. Fibroblast cells were treated with Dulbecco’s Modified Eagle Medium (DMEM) combined with nanobubble solutions, and cell density was measured at 24 and 48 hours. While no significant difference was observed at 24 hours (p=0.52), ozone nanobubbles significantly reduced cell density at 48 hours (p=0.005), indicating cytotoxic effects. Hydrogen and oxygen nanobubble treatments did not show statistically significant differences from the control. These results highlight the cytotoxic effects of ozone nanobubbles on fibroblasts, which may impact their potential application in wound healing. While the study shows the cytotoxic effects of ozone nanobubbles,* in vivo* wound healing and antimicrobial impacts remain unexplored and warrant further study.

## Introduction

Nanobubbles are gas-filled particles with diameters less than 1 μm [1], and their unique properties make them increasingly relevant for biomedical applications such as targeted drug delivery and wound healing [2]. This study serves as an initial exploration into the effects of nanobubbles on fibroblast cells *in vitro*, providing foundational data for their possible clinical applications.

Nanobubbles are characterized by a surrounding layer of electric charges, which allows them to penetrate through microscale gaps between substances and deliver their encapsulated gasses to targeted cells [1]. Due to their negative charge in aqueous solutions [3], nanobubbles are unable to diffuse passively across the negatively charged membrane potential of living cells. Instead, it is presumed that they are transported into the intracellular matrix via endocytosis, similar to the action of nanoparticles [4]. It is noted that positively charged nanoparticles are endocytosed more efficiently compared to their negatively charged counterparts [4]. Once internalized, encapsulated gasses such as hydrogen are expected to influence cellular metabolism and processes such as cell division [5], although the precise mechanisms and effects remain to be fully elucidated. 

The impact of nanobubbles on biological organisms can vary based on the organism type and the specific gas present in the nanobubbles [2,4,6]. However, research on cell proliferation in media that includes nanobubbles remains relatively sparse. Fibroblasts play an integral role in wound healing, as they are involved in extracellular matrix remodeling, tissue repair, scar formation, and collagen production [7]. This study aims to investigate the *in vitro* effects of hydrogen (H₂), oxygen (O₂), and ozone (O₃) nanobubbles on fibroblast cell migration and density using scratch assays, specifically assessing changes in fibroblast cell behavior after exposure to these nanobubbles. It is expected that exposure to H₂ and O₂ nanobubbles will enhance fibroblast migration and proliferation compared to controls, while O₃ nanobubbles will reduce both migration and proliferation due to their toxic properties and potential to induce cell death. This study is limited to examining *in vitro* changes in fibroblast cell density and migration and does not evaluate direct therapeutic applications or effects in wound environments.

## Materials and methods

Cell culture

Fibroblast cells (FC-0024, Lifeline Technologies, USA) were commercially purchased and cultured in high-glucose Dulbecco’s Modified Eagle Medium (DMEM) (Gibco, USA, cat# 2649710), supplemented with 10% fetal bovine serum (FBS) (Gibco, USA). Cells were seeded in 10 cm plates and cultured in a 37°C humidified incubator with 5% CO₂, passaged at 80% confluence.

Scratch wound healing assay

Nanobubble culture mediums were prepared using DMEM powder (Gibco, USA, cat# 12100046) and H₂, O₂, O3 NanoGAS® Water (Shinbiosis Corporation, Osaka, Japan). Culture mediums were filtered through a 0.22 μm membrane filter (AGC Techno Glass Corp., Shizuoka, Japan, cat# 8020-500) to remove pollutants and colloids from the solution.

Passage 9 fibroblast cells were used in the experiment. To examine the effects of nanobubbles on cell migration and growth, the scratch wound healing assay was conducted using the SPLScar™ Scratcher (SPL Life Sciences, Korea, cat# 201906), which has a tip size of 0.5 mm. Fibroblast cells were seeded in 6-well plates at 70-80% confluency. A linear scratch was created across the cell monolayer using the scratch tool, generating a consistent wound size.

Following the scratching procedure, cells were treated with H₂, O₂, and O₃ nanobubble formulations, supplemented with 1% FBS. The control group consisted of cells treated with DMEM and 1% FBS medium only, without nanobubble formulations. All plates were incubated in a 37°C humidified incubator with 5% CO₂ and observed at 24-hour and 48-hour intervals.

Cell counting

To ensure consistency in photographing, the bottom of the 6-well cell culture dish was horizontally marked, aligning the marking with each subsequent photograph. A needle recorder was placed on the microscope stage, and the shooting locations were marked on a sheet spread over the stage to capture the same area across all time points. Photographs of the cells within the scratch area were taken at 0, 24, and 48 hours.

Cell counting was performed manually by analyzing the captured images. The number of cells within the defined scratch area was counted using a calibrated grid in the microscope view to maintain accuracy. Cell density was then calculated by dividing the total number of counted cells by the area of the scratch.

Statistical analysis

Data from cells collected at 24-hour and 48-hour intervals were analyzed to calculate the ratio of each group relative to the control. Cells that exhibited noticeably lower cell density were identified as outliers and removed from the analysis. Statistical significance between nanobubble groups was assessed using the Kruskal-Wallis H test and Dunn’s post hoc test. Statistical significance was defined as p ≤ 0.05.

## Results

**Figure 1:**
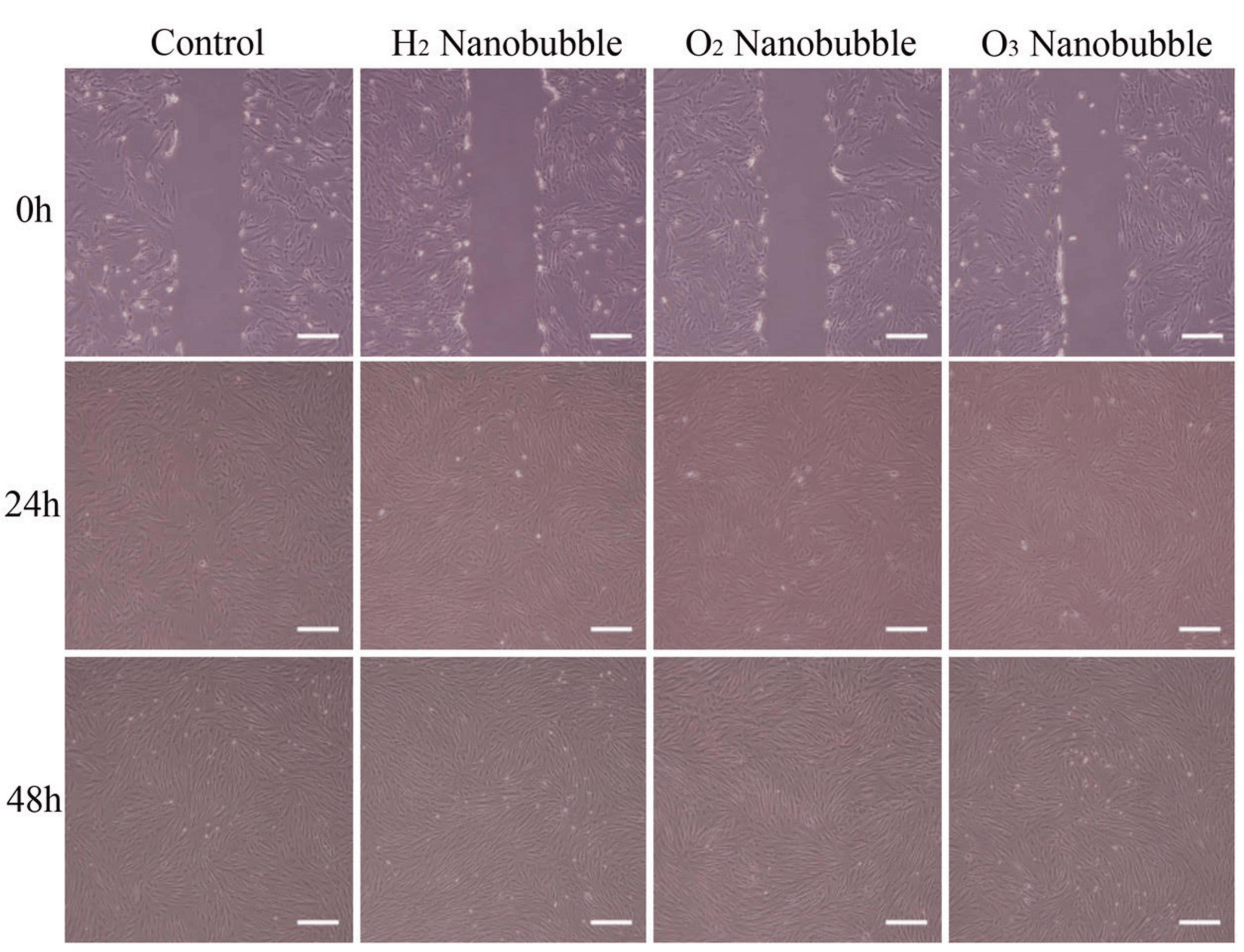
Cell density following scratch assay. The first, second, and third rows show the fibroblast cells at 0 hours, 24 hours, and 48 hours after scratching, respectively. Scale bars: 300 μm

**Figure 2:**
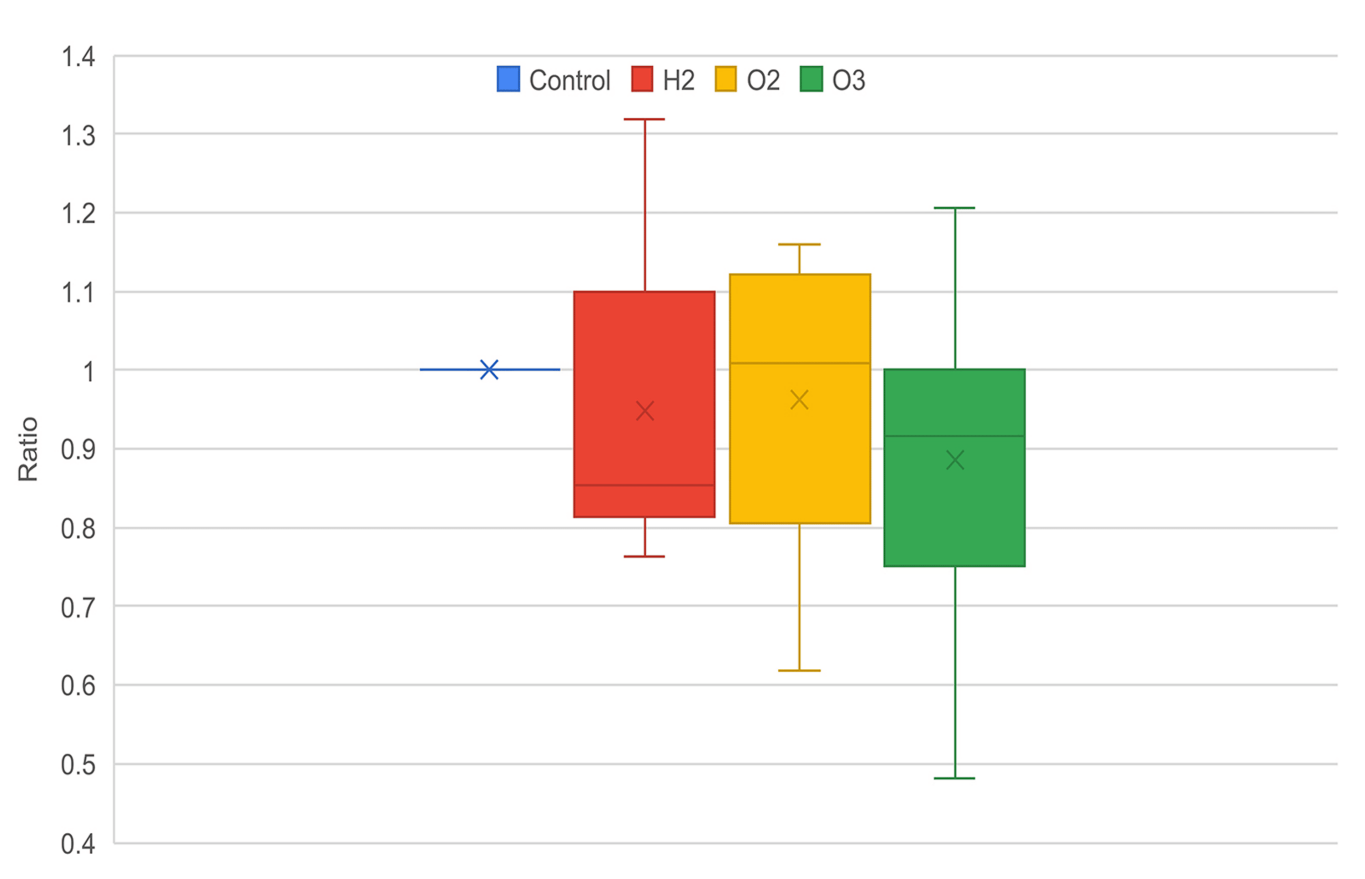
Distribution of cell density ratio against control, 24 hours.

**Figure 3:**
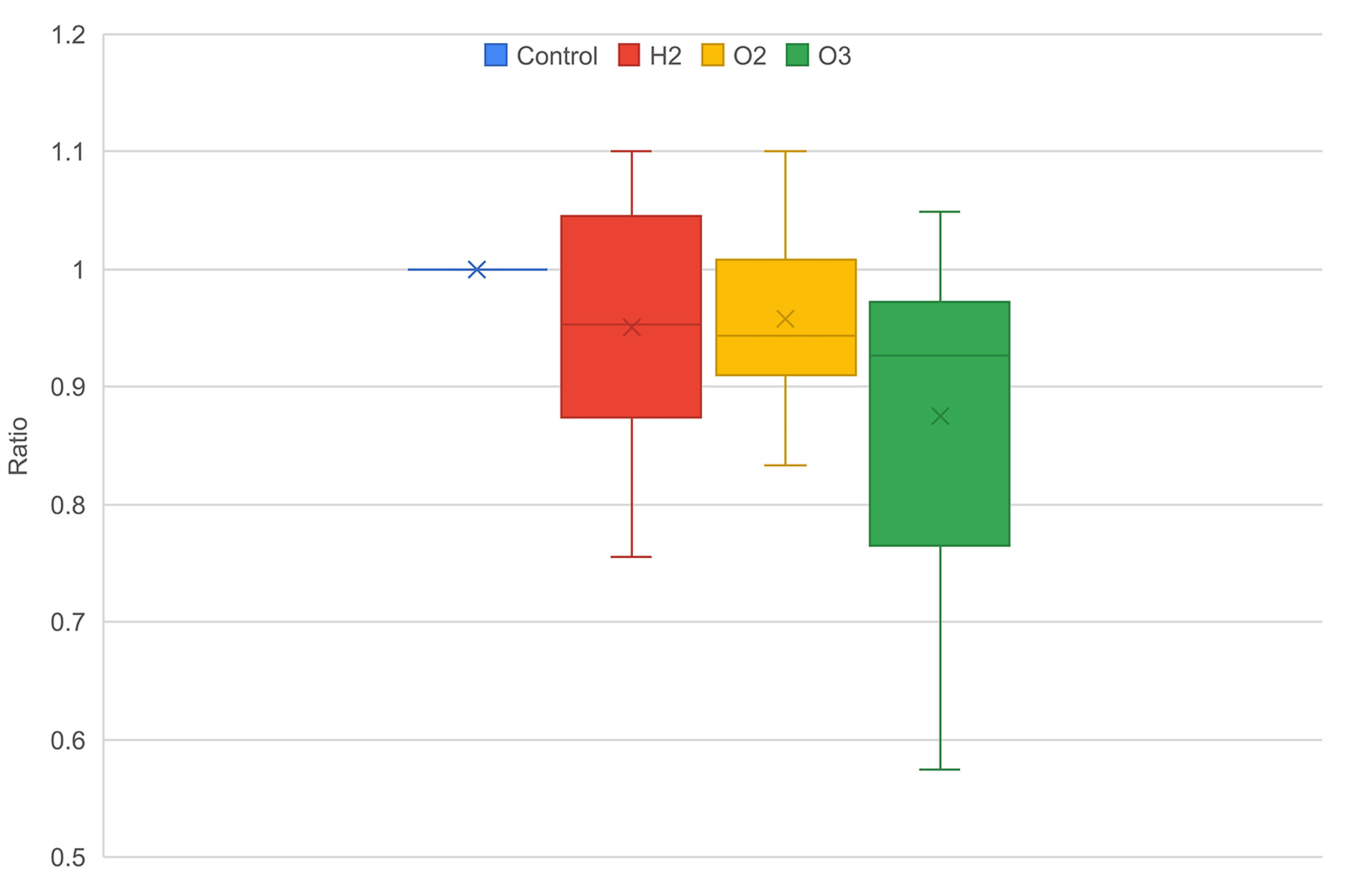
Distribution of cell density ratio against control, 48 hours.

In this paper, we compared the impact of different nanobubble-containing mediums on cell growth and migration through *in vitro* scratch assays (Figure 1). All nanobubble mediums led to a lower average cell density in comparison to the control group after 24 hours and 48 hours (Figures 2, 3). In both time intervals, oxygen, followed by hydrogen nanobubble groups, had similar average cell densities as the control group, with ozone having the lowest cell density. Ozone nanobubbles exhibited the largest standard deviation in both 24-hour and 48-hour groups (Table 1, Figures 2, 3), indicating a higher degree of variability in cell density measurements compared to the other conditions.

A non-parametric Kruskal-Wallis test was used due to the non-normal distribution of our cell density data. The results demonstrate a statistically significant difference between nanobubble groups at the 48-hour interval (p<0.05), though no significant differences were found at the 24-hour interval (p>0.05) (Table 1). This suggests that the impact of different nanobubble types on cell density varied significantly after 48 hours and that the effects of nanobubble treatments may not become apparent until a longer exposure period.

**Table 1:** Intergroup comparisons, Kruskal-Wallis test results.

	24h			48h		
Group	Count	Median	Average Rank	Count	Median	Average Rank
Control	8	1	19.5	9	1	27
H_2_	8	0.855	15	9	0.953	18.6
O_2_	8	1.010	18.25	9	0.943	17.556
O_3_	8	0.917	13.25	9	0.927	12.889
H	2.261			7.959		
p-value	0.52			0.047		

Following the Kruskal-Wallis test, a pairwise comparison using Dunn’s test was conducted on 48-hour data to identify differences between nanobubble treatment groups (Table 2). The comparison between the control group and ozone nanobubbles showed a statistically significant difference (p<0.05), indicating a significantly lower average cell density compared to the control group. The results for the comparisons between the control and hydrogen groups, as well as the control and oxygen groups, showed a tendency towards difference. However, these differences were not statistically significant, with p-values slightly above the 0.05 threshold.

**Table 2:** Post-hoc Dunn's test results, 48 hours.

Group	Test Statistic	Std. Error	Std. Test Statistic	p-value
Control - H_2_	8.4	4.94	1.7	0.089
Control - O_2_	9.44	5.07	1.86	0.062
Control - O_3_	14.11	5.07	2.79	0.005
H_2_ - O_2_	1.04	4.94	0.21	0.832
H_2_ - O_3_	5.71	4.94	1.16	0.247
O_2_ - O_3_	4.67	5.07	0.92	0.357

## Discussion

Nanobubbles refer to extremely small gas bubbles, typically less than 1 μm in diameter, that can remain stable in liquids for extended periods [1]. This study aimed to explore potential applications of nanobubbles in areas such as wound healing, which have not been traditionally associated with these technologies.

Nanobubble size on permeability

Nanobubble size is a critical factor influencing the internalization of their components. Nanobubbles (<1 μm) are hypothesized to facilitate greater cellular uptake compared to microbubbles (1-10 μm). This is attributed to their enhanced ability to penetrate tissues through the fenestrations of the cell membrane [8]. Furthermore, the Noyes-Whitney equation, applicable to particles smaller than 1000 nm, indicates that smaller particles exhibit increased saturation solubility and, consequently, greater bioavailability [9]. Despite the challenges associated with accurately determining nanobubble size [1], it is inferred that a smaller diameter enhances the efficiency of cell permeation.

Mechanism of H_2_, O_2_, and O_3_

Hydrogen gas, due to its nonpolar nature, small molecular size, and low molecular weight, can readily diffuse through cell membranes to exert its biological effects. Ohsawa et al. demonstrated that hydrogen inhalation mitigated ischemia-reperfusion injury in a rat model, suggesting that hydrogen gas alleviates oxidative stress [10]. Reactive oxygen species (ROS) are naturally produced as byproducts of mitochondrial metabolism and are critical for cellular functions, but can cause oxidative stress when their balance is disrupted. ROS are known to regulate the phosphoinositide 3-kinase (PI3K) signaling pathway, which is essential for cell growth, proliferation, and metabolism [11]. Although the precise mechanism of hydrogen gas remains incompletely understood, it is posited that hydrogen may directly influence mitochondrial electron transport [5], thereby neutralizing oxidative stress and restoring ROS balance.

Ozone is widely regarded as having a toxic effect on cells and tissues due to its strong oxidative activity [6]. In this study, we observed a statistically significant decrease in cell density in the ozone treatment group compared to the control group, indicating that ozone may impact cellular migration and growth. While the ozone group showed lower cell densities, the variability in cell density measurements, evident by the larger standard deviation, suggests a complex interaction that warrants further investigation. Despite the observed reduction in cell proliferation associated with ozone exposure, it is crucial to note that cell growth was not completely inhibited. It would be viable to state that while ozone exposure correlated with reduced cell density, the implications for cell survival and functionality remain unclear, as our study did not directly assess cell apoptosis.

Research in other cell types suggests that ozone may influence bacterial growth and cell regeneration [6]; however, our findings are limited to *in vitro* effects on fibroblast proliferation and migration. Any potential impact of ozone nanobubbles on wound healing or antimicrobial activity remains speculative and was not directly assessed in this study. Our findings demonstrated that ozone nanobubbles reduced fibroblast migration and growth *in vitro*. Although ozone’s antibacterial properties could theoretically benefit wound healing by reducing bacterial load, the observed reduction in fibroblast migration and growth indicates a cytotoxic effect. This reduction in cell density may reflect an initial response to ozone treatment rather than a clear advantage for wound healing, particularly in environments challenged by bacterial infection. Given the complexity of wound healing processes involving cell proliferation, various signaling pathways, and immune responses, extrapolating these *in vitro* findings to *in vivo* situations requires caution. While our results demonstrate significant differences in cell density with ozone treatment, evaluations on whether ozone nanobubbles can balance antimicrobial benefits with cytotoxicity in controlled infection models relevant to wound environments would be necessary to further investigate these hypotheses.

Recent research has demonstrated that oxygen nanobubbles can have inhibitory effects on cell proliferation. A study investigating the impact of oxygen and air nanobubbles on dental follicle stem cells (DFSCs) reported a dose-dependent reduction in cell proliferation, with oxygen nanobubbles showing particularly significant effects [12]. This study attributed the observed inhibition to potential oxygen toxicity, suggesting that high oxygen concentrations may alter cellular redox states, creating oxidative stress conditions that are detrimental to cell growth. Similarly, our study observed that ozone nanobubbles, known for their strong oxidative properties, resulted in significant reductions in cell density compared to the control group. Our Dunn’s post hoc test revealed a statistically significant difference between the control and ozone nanobubbles (p=0.005). The pronounced decrease in cell density with ozone nanobubbles aligns with the notion that oxidative stress can negatively impact cellular processes, corroborating the findings of previous studies on oxygen nanobubbles. Our comparison between the control and oxygen nanobubbles showed a p-value of 0.062, which is slightly above the significance threshold of 0.05, yet still indicative of a potential trend toward reduced cell density. The consistency of our results highlights the critical role of oxidative stress in mediating the effects of nanobubbles on cell proliferation, reinforcing the importance of evaluating gas types and concentrations.

Limitations and future directions

This study provides insights into the effects of different nanobubble treatments on fibroblast cell density and migration, particularly in the context of wound healing. By utilizing *in vitro* scratch assays, we have established a foundational understanding of how hydrogen, oxygen, and ozone nanobubbles influence cellular behavior. The statistically significant differences observed in cell density at the 48-hour mark highlight the potential of nanobubble treatments to impact cell proliferation over time, setting the stage for future investigations into their therapeutic applications.

Despite these strengths, the study’s *in vitro* nature is a significant limitation. While the scratch wound assay offers insights into cell migration and proliferation, it does not replicate the complexity of wound healing *in vivo*, where immune responses, extracellular matrix dynamics, and microbial factors play a crucial role in the healing process. These factors are essential to understanding the full impact of treatments on wound healing outcomes. Additionally, the lack of direct measurements of bactericidal activity limits our ability to conclusively claim any antimicrobial benefits of ozone nanobubbles, which were hypothesized but not empirically tested. This absence of data on bacterial reduction means that assertions regarding the potential wound-healing properties of ozone nanobubbles remain speculative. Furthermore, the difficulty in quantifying the exact concentration and stability of nanobubbles in the culture medium over time may affect the reproducibility and consistency of our results. Variability in nanobubble behavior can lead to challenges in interpreting how different treatments influence cellular outcomes.

To address these limitations, future research should focus on directly investigating the wound healing and antimicrobial properties of ozone nanobubbles, prioritizing the bactericidal effects of ozone nanobubbles against common wound pathogens. *In vitro* assessments could determine the effectiveness of ozone nanobubbles in reducing microbial loads in contaminated wound models, followed by exploring their impact on actual wound healing processes through *ex vivo* or *in vivo* models. Understanding how nanobubbles interact with cellular mechanisms during the healing process will provide valuable insights into their therapeutic potential. Moreover, research should aim to identify the optimal concentration of ozone nanobubbles that maximizes antimicrobial activity while minimizing adverse effects on beneficial cell proliferation. Establishing this balance is essential for developing effective wound treatment strategies that leverage the properties of ozone nanobubbles.

## Conclusions

This study explores the potential applications of nanobubbles in influencing fibroblast behavior, particularly in relation to cell density and migration in wound healing models. The results indicate that ozone nanobubbles significantly reduce fibroblast cell density, suggesting cytotoxic effects *in vitro*. Hydrogen and oxygen nanobubbles showed no statistically significant effects on fibroblast cell density compared to controls. These results are specific to fibroblast behavior *in vitro* and do not imply broader impacts on wound healing or antimicrobial activity. Future research should incorporate *in vivo* models and direct antimicrobial testing to better understand the clinical relevance and safety of nanobubbles in wound healing contexts. Any potential wound healing or bactericidal benefits are speculative and require further investigation.
